# An investigation into the factors affecting investigator-initiated trial start-up in Ireland

**DOI:** 10.1186/s13063-020-04893-z

**Published:** 2020-11-23

**Authors:** Lauren Leddy, Prasanth Sukumar, Lydia O’Sullivan, Fionnuala Keane, Declan Devane, Peter Doran

**Affiliations:** 1 UCD School of Medicine, University College Dublin, Dublin 4, Ireland; 2 HRB Trials Methodology Research Network, NUI Galway, Galway, Ireland; 3 HRB Clinical Research Coordination Ireland, Upper Mount Street, Dublin 2, Ireland; 4 National University of Ireland, Galway, Ireland

## Abstract

**Background:**

In common with many countries, Ireland has seen an increasing trend in the number of clinical trials conducted over the past few years. Yet, as elsewhere, trialists in Ireland face several problems and barriers in the starting-up of clinical trials. These barriers impede trial activity significantly, with consequent impacts on patient care. It is critical to understand these issues, to develop approaches to facilitate trial start up. This study identifies the challenges in conducting clinical trials in Ireland and specifically the contractual, ethical, logistical, and regulatory barriers that hinder the start-up of investigator-led trials in Ireland.

**Methods:**

Data for this study were collected in two stages. In the first stage, a survey was conducted among trialists in Ireland. A total of 44 trialists responded to the survey, and information was collected about their experience in conducting clinical trials, the scale and nature of their most recently completed trial, and the details of specific barriers they encountered during the starting-up of the trial. In the second stage, nine semi-structured interviews were conducted with the awardees of 2018 Irish Health Research Board’s Definitive Intervention Feasibility Award. These interviews facilitated a deeper exploration of issues and problems in conducting clinical trials in Ireland.

**Results:**

This study identified several issues and bottlenecks in starting-up clinical trials in Ireland with contracts and ethical approval cited as the major issues. The data shows that site identification and activation was also problematic in some cases. Several respondents reported difficulties in accessing dedicated time for protocol development and believe that support in this area can be greatly beneficial. It was reported that availability of skilled staff members like statisticians and data managers was as an issue, especially for small trials.

**Conclusion:**

This study found that several factors impact trial initiation and progression in Ireland. Delays associated with obtaining contract and ethics approval are perceived as major barriers. Specialist supports in areas such as ethics and regulatory affairs and availability of specialised staff members in areas such as statistics and data management are key actions to enable enhanced clinical trial activity in Ireland.

## Introduction

Clinical trials are an important method for evaluating the safety and effectiveness of health care interventions [1, 2].

However, conducting clinical trials has become increasingly difficult in recent times, irrespective of geographical location or type of trial. Several barriers have been identified, which include declining funding, increasing cost, burdensome regulations, difficulties in patient recruitment and retention, insufficient infrastructure, and lack of skilled personnel [3–6].

Mounting costs, coupled with ever-declining funding, is reported as a serious concern hampering clinical trials [7]. Even though investment in biomedical research shows an increasing trend globally, it is declining in major western developed countries. For instance, federal funding for research and development (R&D) in the USA decreased from USD 126.6 billion in 2010 to USD 120.9 billion in 2017 [8], while in Ireland, Government Budget Allocations for R&D declined from €824.8 million to €739.3 million during the same period [9]. This decline in funding may have particularly detrimental consequences on independent academic clinical research which is often practice-oriented and driven by non-commercial interests [10, 11].

Several recent reports have highlighted the difficulties in conducting clinical research with limited resources and complex regulatory settings [10, 12–16]. Stringent regulations, complex study designs, rigorous monitoring requirements, and an increase in the demand for insurance and indemnification are cited as major factors that contribute to the rising cost of clinical trials [17–19].

The lack of harmonisation in clinical trial regulations between countries and the complexity of these regulations have created substantial delays and cost escalation in setting up multi-country, multi-site studies [10, 20, 21]. This is especially problematic for independent researchers who may not have access to institutional or regulatory specialist support. For instance, it is reported that while it would have taken 6 months and cost around USD 300,000 to initiate a trial over 200 sites in 20 countries in the 1990s, it would now take 4–5 times longer and cost 5–10 times more without any apparent additional benefits [22].

Inadequate access to research infrastructure is cited as another barrier in the successful conduct of clinical trials [12, 23, 24]. Infrastructure, in the form of specialised clinical trial units (CTUs) or clinical research centres (CRCs), can provide investigators with the necessary support in grant application preparation, developing research design and methodology, and assistance in securing regulatory and ethical approvals [25, 26]. However, many independent academic investigators may not have access to these types of facilities, and this prevents them from getting involved in clinical trials.

In Ireland, there was a 34% increase in the number of sites conducting clinical trials since 2014 [27]. An analysis of the number of trials registered on ClinicalTrials.gov shows that approximately 500 interventional clinical trials have been active in Ireland in the past 5 years. It is likely that trialists in Ireland will face similar challenges in conducting clinical trials to those outlined above. Identification and recognition of these challenges may provoke suggestions and actions to rectify the current clinical trial process in Ireland. The aim of this study is to identify the overall challenges in conducting clinical trials in Ireland and specifically the contractual, ethical, logistical, and regulatory barriers that hinder the start-up of investigator-led clinical trials in Ireland.

## Data and methods

Data collection for this study comprised of two stages. In the first stage, an online survey was conducted among trialists in Ireland to collect information on the major hurdles they encountered in study start-up. In the second stage, nine semi-structured telephone interviews were conducted with the 2018 awardees of Irish Health Research Board’s (HRB) Definitive Intervention Feasibility Award (DIFA). The HRB DIFA award is a major funding scheme available to senior level researchers in Ireland to evaluate full-scale definitive interventions and feasibility studies. Awardees of this scheme are usually well-experienced senior researchers, and interviews with them will help to gain an in-depth understanding of the issues and problems they face in the conduct of clinical trials in Ireland.

### Stage 1: The online survey

The survey had three main sections. Part one focused on the background of the respondents and extracted information regarding their experience and prior involvement in clinical trials. Part two captured basic data about the scale and nature of the respondents most recently completed clinical trial. It also collected information on timeframes for processes such as ethical approval and site selection. Part 3 looked specifically at any barriers encountered in staff availability, protocol development, funding and budget, statistics and data management, supplies, clinical operations, contracts, and regulatory strategy. The online survey was developed and implemented using the REDCap [28] data capturing tool hosted on University College Dublin (UCD) Information Technology (IT) systems.

A list of 232 currently active clinical researchers based in seven University Clinical Research Facilities/Centres and their associated hospitals in Ireland in 2018 was obtained from HRB Clinical Research Coordination Ireland, and the online survey was sent to them via email. This included Research staff (including research nurses and coordinators) and investigators.

### Stage 2: Semi-structured interviews

Telephone interviews were conducted with nine investigators in receipt of the DIFA for 2018. An interview schedule was used to guide the interview process. The interview followed the same structure of the survey questionnaire; however, the questions asked were open-ended. This provided the opportunity to gather more detailed and richer information enabling a deeper exploration of each topic. The interviews lasted typically 20 min and were conducted based on the participant’s availability.

### Data analysis

Survey data were exported to statistical software package SPSS [29] for analysis. Descriptive statistics (proportion) was used to present the results.

LL conducted the interviews over the phone. Telephone interviews were recorded, transcribed, and then exported to qualitative data analysis software Nvivo (version 12) for analysis. PS, an experienced social scientist, led the analysis. Data were analysed thematically to identify patterns in the data [30]. LL and LOS reviewed the codes and themes and agreement and disagreements discussed until a consensus reached.

## Results

### Survey results

#### Characteristics of the respondents

A total of 44 researchers responded to the survey (response rate 19%). Table 1 presents details of respondents’ professional background and research experience.

**Table 1:** Respondents’ background

	***n***	%
**What type of investigator are you?**		
Principal Investigator	10	22.7
Sub-Investigator	3	6.8
Research Nurse	18	40.9
Study Coordinator	4	9.1
Other	9	20.5
**Where do you do your research?**		
Hospital	15	35.7
Clinical Research Centre	25	59.5
Other	2	4.8
**Do you have access to?**		
Clinical Research Centre / Facility	36	97.3
Hospital Research Office	10	27.0
University Research Office	11	29.7
**Number of clinical trials currently involved**		
0	5	12.2
1–3	21	51.2
4–6	9	22.0
7–9	4	9.8
> 10	2	4.9
**Number of clinical trials involved in past 5 years**		
0	4	9.8
1–5	16	39.0
6–10	12	29.3
11–15	3	7.3
> 16	6	14.6
**Who negotiates/agrees budgets?**		
Hospital	3	7.5
University	19	47.5
Other	18	45.0
**Who negotiates /approves contracts?**		
Hospital	14	29.2
University	19	39.6
Other	8	37.5

Most of the respondents who participated in the survey were ‘research nurses’ (*n* = 18, 40.9%) followed by Principal Investigators (‘PIs’) (*n* = 10, 22.7%). ‘Sub-investigators’ (*n* = 3, 6.8%) and ‘study coordinators’ (*n* = 4, 9.1%) also responded to the survey.

Almost 90% of the respondents reported ongoing clinical trial activity with a cumulative involvement in 142 trials (range 1–15, median 3). In addition, over 90% reported involvement in clinical trials in the past 5 years, with a cumulative involvement in 389 trials (range 1–80, median 6). This shows that some of the survey respondents had significant experience in performing clinical research. However, about 12% (*n* = 5) reported that they were not involved in any clinical trials at the time of the survey and roughly 10% (*n* = 4) reported no involvement in the past 5 years.

When asked about where they primarily conduct their research, almost two thirds (*n* = 25, 59.5%) answered ‘Clinical Research Centres’ and one third reported hospital-based research. Most respondents stated that they had good access to various supports to facilitate their research. In response to a question about their availability and access to facilities, 97.3% (*n* = 36) reported that they had access to ‘Clinical Research Centres’, 29.7% (*n* = 11) had access to a ‘University Research Office’, and 27% (*n* = 10) had access to a ‘Hospital Research Office’. This shows that the majority of the survey participants receive good institutional support.

To explore the extent to which the respondents’ affiliated institutions facilitated their research, two questions were asked about who would negotiate/agree on contracts and budgets for their studies. Slightly more than half (55%, *n* = 22) of the respondents reported that their institution (university or hospital) was involved in negotiating/agreeing on budgets. However, in the case of contracts, this figure was about 70% (*n* = 33).

#### Experience in most recently completed trial

Survey participants were presented with several questions regarding their trial experience, focusing specifically on their most recently completed trial. These questions included start and end dates of the trial, type of trial, the time taken to obtain ethical and regulatory approvals, staff availability, and other issues and barriers at different stages of the project. Table 2 presents the details of the most recently completed trial respondents were involved with.

**Table 2:** Information about the most recently completed trial

	***n***	%
**Phase of the trial**		
Phase 2	4	21.1
Phase 3	10	52.6
Phase 4	2	10.5
No answer	3	15.8
**Type of trial**		
Academic	8	42.1
Commercial	11	57.9
**Was it?**		
Multi-centre	16	88.9
Multinational	15	78.9
**Total time taken for study start-up**		
< 4 weeks	1	5.3
1–6 months	9	47.4
6–12 months	3	15.8
1–2 years	5	26.3
2–3 years	1	5.3
**Time taken for site selection**		
< 4 weeks	2	13.3
1–6 months	8	53.3
6–12 months	2	13.3
1–2 years	1	6.7
2–3 years	2	13.3
**Time taken for ethical approval**		
< 4 weeks	1	5.6
1–6 months	14	77.8
6–12 months	2	11.1
1–2 years	1	5.6
**Did the study complete on time?**		
No	6	40.0
Yes	9	60.0
**Did the study complete on budget?**		
No	1	6.7
Yes	14	93.3

Only 14 survey participants reported the start and end dates of their most recently completed trials. Of these, 20% (*n* = 3) reported that their most recently completed trial was completed in 1 year, whereas 50% (*n* = 7) of the respondents reported that it took 1–2 years to finish. In the case of about 30% (*n* = 4) of the respondents, their most recent trial continued for more than 2 years.

Of the 16 participants who responded to a question about the phase of their most recently completed trial, the majority (62.5%, *n* = 10) reported that the trial was a phase 3 trial. About a quarter (*n* = 4) reported that their most recently completed study was a phase 2 trial and 12.5% (*n* = 2) reported it was phase 4 trial. Interestingly, no one reported that they had conducted a phase 1 trial recently.

Further questions were asked about the time taken to select study sites and obtain various approvals necessary for the initiation of the trial. In most cases, these approvals were received within 1–6 months (Table 2). However, almost one third of the respondents reported that site selection and contracting took more than 1 year to complete. When comparing the approval time of academic studies with commercial studies, it seems that commercial studies take comparatively less time to set-up than academic studies (Table 3). In the case of almost 80% (*n* = 14) of participants, ethical approval for the study was received within 1–6 months.

**Table 3:** Time to get approvals: academic vs. commercial trials

Approval time	Academic		Commercial	
	*n*	%	*n*	%
< 4 weeks	1	12.5	0	0.0
1–6 months	2	25.0	7	63.6
6–12 months	1	12.5	2	18.2
1–2 years	3	37.5	2	18.2
2–3 years	1	12.5	0	0
Total	8	100	11	100

It was reported that the time taken to select sites and get various approvals impacted the overall study start-up time. Approximately half of the respondents (*n* = 10) reported that they could commence their most recently completed trial in less than 6 months. For about a quarter of the respondents (*n* = 5), it took 1–2 years from initial contact to study start-up. For a minority of the respondents (5%, *n* = 1), it took 2–3 years to study start-up.

The delays in getting various approvals and study site selection might have affected the overall study completion time. As shown in Table 2, only 60% (*n* = 6) of the respondents reported that they could complete the study on time. However, funding was not reported as a problem. The majority (90%, *n* = 14) reported that they could finish the study on budget.

#### Barriers to study start-up

Several questions were asked about barriers respondents faced in their most recently completed trial. These included the availability of staff, issues in the development of protocol and budget, statistical and data management issues, problems with supplies, and issues in clinical operations.

Table 4 shows the survey participant’s responses to the questions on the availability of staff for the study. The majority reported that the availability of research nurses and sub-investigators was not a problem; however, many reported that the availability of data managers (27%, *n* = 5), statisticians (27%, *n* = 5), and research pharmacists (16%, *n* = 3) was problematic during their study.

**Table 4:** Staff availability

	***n***	%
**Sub investigators**		
No	3	16.7
Yes	14	77.8
Not applicable	1	5.6
**Research nurse**		
No	1	5.6
Yes	14	77.8
Not applicable	3	16.7
**Data manager**		
No	8	44.4
Yes	5	27.8
Not applicable	5	27.8
**Statistician**		
No	8	44.4
Yes	5	27.8
Not applicable	5	27.8
**Research pharmacist**		
No	10	55.6
Yes	3	16.7
Not Applicable	5	27.8
**Any issues writing protocol**		
No	3	20.0
Yes	3	20.0
Not Applicable	9	60.0
**Any changes required in protocol**		
No	5	35.7
Yes	4	28.6
Not Applicable	5	35.7

About 20% (*n* = 3) of respondents reported that they encountered issues when writing the protocol and about 28% (*n* = 4) noted the need to alter the protocol as the trial progressed.

Table 5 shows the details of the funding received and financial management-related issues for the most recently completed trial. The majority of the most recently completed studies (*n* = 7) were medium size studies with less than €100K funding, and 8.3% (*n* = 1) of the studies were large studies with more than €3 Million funding. Most of the respondents (75%, *n* = 9) reported that the funding they received was adequate for the study.

**Table 5:** Funding and financial management

	***n***	%
**Cost analysis prior to the study?**		
No	1	8.3
Yes	9	75.0
Not applicable	2	16.7
**Coverage analysis prior to the study?**		
No	0	Nil
Yes	11	91.7
Not applicable	1	8.3
**Financial monitoring throughout?**		
No	2	16.7
Yes	8	66.7
Not applicable	2	16.6
**Funding received**		
No funding or unknown	3	25.0
< 10,000	2	16.7
10,000–50,000	3	25.0
50,000–100,000	2	16.7
100,000–500,000	1	8.3
> 3,000,000	1	8.3
**Was funding adequate?**		
No	0	0.0
Yes	9	75.0
Not Applicable	3	25.0

Financial management was not an issue for most trialists. The majority reported that cost analysis (75%, *n* = 9) and coverage analysis (91%, *n* = 11) of the study were carried out prior to the commencement of the trial. About two thirds (*n* = 8) reported that financial monitoring of the study was continued throughout the study period.

Respondents were asked about problems associated with statistics and data management (Table 6). A minority of respondents (< 10%, *n* = 1) recalled issues in this area, apart from the database design, which was highlighted as a problem by 16% (*n* = 2) of respondents.

**Table 6:** Issues in statistics and data management

	*n*	%
**Statistical design**		
No	5	41.7
Yes	1	8.3
Not applicable	6	50.0
**Sample size calculation**		
No	5	41.7
Yes	1	8.3
Not applicable	6	50.0
**Statistical analysis plan**		
No	5	41.7
Yes	1	8.3
Not applicable	6	50.0
**Case report form design**		
No	8	66.7
Yes	1	8.3
Not applicable	3	25.0
**Database design**		
No	5	41.7
Yes	2	16.7
Not applicable	5	41.6
Interactive voice system^*^		
No	7	58.3
Yes	1	8.3
Not applicable	4	33.3
**Data entry**		
No	9	75.0
Yes	1	8.3
Not applicable	2	16.7

*Automated system for randomisation

The respondents were asked about issues in sourcing various suppliers for their trial (Table 7). As the type of study intervention can influence the supplies required, a question was asked firstly about the type of intervention. Trials on ‘approved investigational medicinal products (IMP)’ (41%, *n* = 5) and ‘unapproved IMP’ (41%, *n* = 5) were reported as the main type of intervention. About 8% of the respondents reported that the intervention in their trial was ‘diagnostic strategies’ (*n* = 1) and the same number stated that their trial was a procedure like a surgical intervention.

**Table 7:** Issues with supplies

	***n***	%
**What type of intervention**		
Approved IMP	5	41.7
Unapproved IMP	5	41.7
Procedure (e.g. surgical)	1	8.3
Diagnostic Strategies	1	8.3
**Any issue in sourcing supply**		
No	10	83.3
Yes	1	8.3
Not applicable	1	8.3
**During labelling and packaging**		
No	6	50.0
Yes	1	8.3
Not applicable	5	41.7
**During destruction**		
No	8	66.7
Yes	0	0.0
Not applicable	4	33.3

Respondents were asked about issues they confronted during sourcing, labelling/packaging and destruction of the supplies. A small proportion of respondents (8.3%, *n* = 1) reported that they had encountered any issues during the sourcing and labelling/packaging of the supplies. However, no one reported any issues with the destruction of the supplies.

Questions about bottlenecks in clinical operations were also asked. These included issues in site identification, site selection, site activation, and study monitoring (Table 8). No one reported site identification or site selection as an issue. However, a few (8.3%, *n* = 1) reported that site activation was problematic and about 17% (*n* = 2) cited study monitoring as a concern.

**Table 8:** Issues with clinical operations

	*n*	%
**Time between site identification and activation**		
< 4 weeks	2	16.7
1–6 months	5	41.7
6–12 months	3	25.0
1–2 years	1	8.3
2–3 years	1	8.3
**Any issues during site identification?**		
No	11	91.7
Yes	0	0.0
Not applicable	1	8.3
**Any issue with site selection**		
No	10	83.3
Yes	0	0.0
Not applicable	2	16.7
**Any issue with site activation**		
No	10	83.3
Yes	1	8.3
Not applicable	1	8.3
**Any issue in study monitoring**		
No	9	75.0
Yes	2	16.7
Not applicable	1	8.3
**Any issues in contract negotiation**		
No	9	75
Yes	3	25
**Duration of contract execution**		
1–6 months	9	75.0
6–12 months	2	16.7
> 1 year	1	8.3
**Issues with ethical approval**		
No	9	75
Yes	3	25
**Issues with regulatory approval**		
No	11	91.70
Yes	0	0
Not applicable	1	8.30

A quarter of respondents (*n* = 3) reported complications with contract negotiation, while 75% reported they never had any issues in this area. The majority reported that the contract execution finished in less than 6 months, but for a few (8.3%, *n* = 1), this took 1–2 years.

Finally, questions were asked about any problems encountered during the ethical and regulatory approval. No participants reported issues during regulatory approval, while a quarter of the respondents reported that they had issues in receiving ethical approval in time.

### Results of qualitative interviews

Of the 10 recent DIFA awardees, nine were interviewed over the course of this project. Interviewees were an experienced group of trialists with 57 trials currently ongoing (range 1–30) and 93 trials completed in last 5 years (range 1–45). All of their ongoing studies were multicentre trials, with 36% of trials being multinational in design.

In the interviews, the DIFA awardees were presented with several open-ended questions about their experience in starting-up their currently active studies. Analysis of the interview data highlighted barriers to study start-up that grouped into four thematic areas, i.e. budget and funding, staff and institutional support, site identification and selection, and ethical and regulatory issues. Analysis of the interviews largely reflected the findings of the online survey. Very similar barriers and issues were reported in both the survey and the interviews.

#### Budget and funding

There were several issues relating to budget and funding. Although nearly all interviewees stated that the amount of funding was adequate for the study, a few recalled unanticipated costs which generated problems in the budget plan. For example, some interviewees reported that the unexpected salary raises required in accordance with the agreement between the Government and the Public Service Unions had affected their budget plans.

Changes to the budget plan have already been made however due to Haddington Road Agreement – increased salaries therefore money had to be moved around to accommodate this. (PI-1)

Adequate until government raised salaries last year but HRB didn’t take this into account – needed to revise budget to reduce staff time slots as salaries increased. (PI-3)

To adjust for this increased staff cost, staff time had to be reduced, which may have affected the overall study progress. There were also instances of unexpected higher insurance costs which prompted PIs to look for funding from other sources. One PI reported that the funding was so tight that they were unable to avail of legal resources to put contracts through which resulted in delays in the overall study start-up.

In general funding is extremely tight for academic studies. This has implications downstream for example don’t have legal resources to put contracts through, this results in delays. (PI-5)

Insurance has caused additional costs that were not budgeted for, currently looking to source more funds. (PI-2)

Only a very small proportion of the respondents reported any overspending, and in that case, this was mostly on IT resources and training.

#### Staff and institutional support

Staff availability was generally not cited as an issue; however, one PI raised concern over the lack of availability of experienced statisticians.

Noted availability of an experienced statistician is huge area of deficiency in academic studies in comparison to other countries. (PI-5)

Another interviewee highlighted the importance of staff need to be trained in other aspects of clinical trial including data management, trial management etc.

Dr [name] first time involved in research and felt there were lots of additional requirements involved in getting a clinical trial up and running. Suggested general training prior to trial start up for new investigators on areas such as (staff needed in areas such as data management, statistics etc.). She noted that it is research plus trial management and would like training on how best to go about organising both these aspects of clinical trials simultaneously. (PI-1)

The majority did not report any problems with the protocol development stage. However, a few reported that lack of protected time for research activities and inadequate support from experienced staff affected protocol development.

No protected time. Currently no research fellow therefore PI has extra responsibility. Things have changed since initially written caused delays. Introduction of GDPR was big problem. (PI-7)

Sourcing external help, for instance, assistance from a regulatory affairs manager, was cited as very useful in the preparation of the protocol. No major issues were raised regarding sourcing, labelling/packaging, and destruction of the supplies. However, two respondents recalled that there were significant delays in the sourcing of the supplies mainly due to the cumbersome tendering processes required.

Always issues and delays as ‘people don’t deliver’ however the other delays regarding procurement of the intervention superseded these delays. (PI-8)

#### Site identification and selection

Some participants reported that there were several problems in site identification, selection, and initiation. In some cases, multiple site visits were required to assess the site feasibility. Lack of timely response from the staff at prospective sites was also an issue that resulted in delays.

The biggest problem is with a site in [place]. The quality manager wants to carry out own ethics despite having received national ethics approval. The contract review process takes 6 months which means delays on site starting. These delays have been very disappointing. (PI-4)

In some cases, agreeing contracts with the sites was reported lengthy and problematic. In one case, the contract had to be revised due to the newly implemented General Data Protection Regulation (GDPR).

Had to be revised (the contract) due to issues with GDPR, data processing and data transfer agreements. (PI-7)

Contracting very delayed, has already taken one year and is ongoing. (PI-2)

#### Ethical and regulatory issues

In addition, delays with ethics approval were noted. In one case, this was due to administrative delays and staff change-over at the ethics committee. Another awardee mentioned that the requirement for multiple approvals from different ethics committees hindered trial progression.

Ethics in hospitals in general are the main problem. The need to receive ethics approval from each hospital can take months … ... Should be standardised national ethics form which is used at every site. (PI-3)

## Discussion

This study aimed to identify the barriers clinical trial investigators face in the initiation and completion of clinical trials in Ireland. It is recognised that a team effort is essential to ensure success in clinical research and thus a varied experience base and insight was welcomed in stage 1 of the study. In stage 2, the interviews were designed to specifically target the ten 2018 DIFA awardees. These participants were active PIs in Ireland and their opinions were regarded highly as a reflection of the current state of clinical trials in Ireland in 2018.

Below, the findings of the most prominent hurdles are compared, as highlighted by each data set, to published findings in other countries.

### Contracts

The majority of interviewees experienced problems with negotiation and agreement of contracts both with funders and study sites. The survey data show that ‘contracting’ was most often negotiated and agreed at the institution level. Almost one third of the survey participants reported that the contracting process had taken 1–3 years. Major bottlenecks mentioned by the interviewees included GDPR restrictions, difficulty in organising all personnel involved (trial staff, lawyers etc.), lack of communication between parties, and delays in communication.

### Ethical approval

Delays associated with receiving ethical approval were also highlighted in both the survey and the interviews. These results concur with a recent work which reported that complex regulatory requirements delayed the recruitment to their trial in 57 sites across 16 countries by an average of 14.88 months in non-US sites and 12.08 months in the US sites [20]. Another report found that a phase III cancer therapeutic trial in the US had required 769 steps, 36 approvals, and approximately 2.5 years to open the study [14]. Several authors agree that delays such as these can contribute to research waste [6].

A pilot scheme in Ireland [31] which combines the ethics and regulatory clinical trial application into a single step may prove effective in reducing these delays. It is hoped that this will provide the impetus and resources to implement efficiencies in the ethical review and approval process for clinical trials. Recently, the UK has introduced model clinical trial agreements [32, 33] for both commercial and non-commercial trials, and it is hoped that this may speed up the approval process. Ireland may also benefit from the introduction of standardised clinical trial agreement templates which can speed up the trial initiation.

### Protocol development

The online survey found that a small proportion of respondents encountered difficulty in writing the protocol and a few had to amend the protocol during the trial. Protocol amendments may have affected the study progress; as reported by Lamberti et al. [34], a substantial amendment increased the study duration by 3 months on average. One suggested solution for these kinds of issues was engaging with a registered clinical trial unit with a dedicated regulatory affairs manager early in the development of protocols, which might be beneficial in addressing the difficulties in protocol development and subsequent amendments.

### Budgetary and funding considerations

Survey and interview data suggest that, in most cases, funding received for the study was sufficient and financial management was well conducted throughout. All DIFA awardees who participated in the interviews reported that they were responsible solely for the budget development and negotiation for their studies. This was also reported in the online survey. Analysis of the qualitative data revealed that issues with insurance and mandatory staff salary raises created issues with financial management. This suggested that perhaps consultation with financial professionals or increased institutional involvement in this area may provide more support to PIs. An interesting finding is that none of the respondents in this study reported their funding as ‘inadequate’. In contrast, a global survey among academic cancer researchers found that lack of funding was the most reported barrier in cancer research [35]. Similarly, the UK [36] and Australian [37] surveys mentioned above cited time and money deficits as some of the biggest roadblocks to clinical research. In Ireland, there can be significant variation in the funding available to clinicians and other health care professionals, depending on, for example, the availability of charitable support at a local level or whether researchers can access grants allocated for a specific disease area. It should also be pointed out that all of the interview participants were recipients of a DIFA grant. Therefore, it is possible that if this study was repeated with a larger sample size, more respondents would have reported finances as a barrier.

### Site identification, selection, and activation

The interview data suggest that site activation was problematic in some cases. Several issues were raised as the reason for delayed site activation, which include, multiple site visits being required, delayed responses from site staff, etc. Studies have already reported that site activation is an issue [38], which not only delays the trial progression, but is also a waste of resources.

### Staff availability

Having a trained and skilled team to conduct trials is important for the successful completion of clinical trials. The survey found that the availability of staff such as sub-investigators and research nurses was generally not a problem. However, availability of other types of skilled staff members like statisticians and data managers was reported as an issue, especially for small trials. This resulted in these tasks being delegated to sub-investigators who may not have been skilled in this area, resulting in delays. Given the importance of prospectively designing the statistical plan within clinical trials and also maintaining data integrity, it is concerning that these specialist staff were not available to researchers.

In the survey, a number of respondents (*n* = 6) reported that issues related ‘Statistical Design’ and ‘Statistical Analysis Plan’ were ‘Not Applicable’ to them. A further review of the characteristics of these respondents who reported ‘Not Applicable’ to this question show that they were mostly research nurses. This response is not surprising considering the role of research nurses in the clinical trial process as they are mostly focused on the collection of study outcomes.

### Limitations of this study

Poor response rate is a major limitation of this study. A larger sample size may have revealed additional perspectives, particularly relating to finances. Disproportionate representation of particular professional groups like research nurses in the survey might have skewed the findings of the study. Poor response rate from other professional groups, especially clinicians, might be due to time constraints or lack of interest. The funding levels for the most recently completed trials suggest that only a few of them were large multicentre trials. Large multicentre trials may experience different issues and low representation of them is another limitation of this study.

Only a small number of respondents reported any problems with sourcing, packaging, or labelling of the investigational product, and there were no issues cited with drug destruction. However, it is not clear whether any of the respondents were pharmacists. The clinical trials’ pharmacist plays a crucial role in the safe preparation, labelling, and storage of the investigational drug, in addition to contributing to the protocol design, educating patients regarding medication adherence and ensuring ongoing protocol and GCP compliance. Therefore, insights from this important member of the research team may have been missed [39].

Only less than half of the respondents attempted the parts 2 and 3 of the survey which is another major limitation of the study. Comparatively more principal investigators, sub investigators, and study coordinators (~ 50%) completed parts 2 and 3 of the survey, while only about one third of research nurses completed these sections. Many research nurses may not have much detailed information about every aspect of their most recently completed study which might be the reason for their low response rate to these sections.

## Conclusion

The data on the number of clinical trials being conducted in Ireland suggests lower trial participation in Ireland in comparison to other European countries. This study has attempted to uncover the prime barriers of trial hindrance, as highlighted by active trial staff whose opinions are rooted in experience. It appears that many factors impact trial initiation and progression; however, delays associated with contracting and ethics in particular are perceived as the major barriers to investigator-initiated trial start-up in Ireland. Additional specialist supports such as ethics and regulatory affairs and statistics and data management may enhance the quality and efficiency of clinical trial activity in Ireland. Similar studies conducted in other countries suggest that the issues and barriers identified by this study are not unique to Ireland.

Since 2014, the Health Research Board Clinical Research Coordination Ireland (HRB CRCI), a network of 7 University Clinical Research Facilities/Centres and their associated hospitals in Ireland, provides centralised clinical research support for both commercial and academic clinical trials in Ireland. The Clinical Research Facilities and Centres support researchers by providing them with necessary infrastructure and specialist staff support. Although almost all respondents participated in the survey reported that they had access to Clinical Research Centre/Facility, the findings from this study reveals that these facilities need to be further strengthened and streamlined to better cater the needs of the research community in Ireland.

## Data Availability

The datasets used and analysed during the current study are available from the corresponding author on reasonable request.
